# Guidelines, guidelines and more guidelines: And we still do not know how to follow-up patients with breast cancer

**DOI:** 10.1186/1477-7819-3-54

**Published:** 2005-08-23

**Authors:** Steven D Heys, Shailesh Chaturvedi, Andrew W Hutcheon, Tarun K Sarkar

**Affiliations:** 1 Section of Surgical Oncology, Department of Surgery, University of Aberdeen, Medical School, Foresterhill, Aberdeen, Scotland, AB25 2ZD, UK

## Abstract

**Background:**

A major challenge facing us is the provision of health care and appropriate allocation of available resources for the treatment of patients with breast cancer. This is of particular concern in the provision of follow-up care. With the increasing incidence of breast cancer and the improvements in survival which have resulted in up to 75% of patients surviving for more than five years, an increasing resource is required. However, there is controversy as to the most appropriate schedule for follow-up of these patients. This brief review has focused on the evidence-base and guidelines that currently exist in the United Kingdom for the follow-up of patients who have been treated for breast cancer.

**Methods:**

A review of the current guidelines published in the United Kingdom for the follow-up of patients with breast cancer (National Institute for Clinical Excellence, Scottish Intercollegiate Guidelines Network, British Association of Surgical Oncology) and the randomised controlled trials evaluating the follow-up of patients with breast cancer was undertaken.

**Results:**

The results have demonstrated the different follow-up protocols currently indicated in these guidelines within the same country. Furthermore, the lack of well designed, randomised controlled trials on which to base a follow-up protocol for patients with breast cancer is apparent.

**Conclusion:**

The evidence-base on which these guidelines have been developed is lacking. It is apparent that well designed randomised controlled trials are needed urgently if we are to understand the most appropriate and effective ways of following up patients with breast cancer.

## Background

A major challenge in the provision of healthcare throughout the world in the 21^st ^century is trying to ensure that the resources that are available meet the demand. Nowhere is the situation more acute than in the provision of care for patients with cancer. Whilst current statistics show that one in three people in the world will develop a malignant disease, this figure is the projected to increase dramatically during the next 10 years.

Of particular concern has been the continual rise in the incidence of breast cancer. Each year the incidence increases by approximately 2% and in the UK alone there are 45,000 new cases per annum [1]. However, whilst the incidence of breast cancer is increasing there have been many improvements and developments in surgery, radiotherapy, chemotherapy and hormone therapy for patients with breast cancer [2]. The resultant improvements in survival are well recognised, and in Scotland, for example, the five year survival has risen now to 75% [3]. These improvements in survival are most welcome but we do need to consider the utilisation and allocation of resources for the follow-up of these patients and their concerns as to their appropriate use.

Therefore, how should we follow-up patients with breast cancer who have undergone apparently curative therapy? Firstly, the natural history of the disease, i.e. the probability of local and/or distant recurrence of disease and the psychological morbidity must be considered in this regard. Secondly, the short-term and longer-term effects on the patient of the various treatments that they have been given (surgery, radiotherapy, chemotherapy, hormone therapy) must also be taken into account. For example after surgery, wound complications, postoperative pain, lymphoedema and disorders of body image occur. After chemotherapy and hormone therapy, there is risk of cardiac dysfunction, neurotoxicity, premature menopause, osteoporosis, osteoporotic fractures and psychosocial disturbances that must be taken into account. Furthermore, the possibility of a new primary cancer in the ipsilateral, or contralateral, breast is also important.

In terms of the disease itself, approximately 25% of patients will develop a systemic recurrence and die within five years. Importantly, 60% to 80% of all recurrences that occur are found during the first 3 years after treatment of the primary tumour in the breast [4,5]. Furthermore, in approximately three quarters of patients who develop disease recurrence, there are symptoms experienced by the patient or there are abnormalities on clinical examination to indicate recurrence of disease [4]. The risk of local disease recurrence in a breast which has been treated by breast conservation surgery, or the risk of a second primary breast cancer occurring in either breast, is approximately 0.5% to 1% per annum, every year, following completion of treatment [5].

Given this information, follow-up care for patients with breast cancer has been directed primarily towards detecting local and lymph node recurrence of disease and also with the aim of detecting metastatic disease using clinical examination and radiological and laboratory tests. However, three key questions should be considered when planning a follow-up programme for patients with breast cancer:

• How effective are regular hospital visits, clinical examination and laboratory investigations in detecting disease recurrence, and how effective is mammography in identifying local recurrence or second primary cancers in the breast?

• If disease recurrence (local, regional systemic) is identified can the patients' outcome in terms of survival be altered?

• If there is an effect on patients' outcome and survival, then what is the optimal schedule of investigations in order to achieve this?

### What is the evidence?

A major limitation in trying to answer these questions is the quality of the evidence that is available. Whilst there are many retrospective and prospective observational studies of follow-up of breast cancer patients, these are all open to a variety of biases, which severely limit the interpretation of these observations. The only way in which we can answer the questions about follow-up is through well designed, adequately powered and well conducted clinical trials. At present there are few randomised controlled trials in the follow-up of patients with breast cancer that are available.

However, firstly can we affect the outcome of patients by detecting recurrent disease at an early stage by using an intensive follow-up schedule? In order to answer this question, two trials have examined more than 2,500 patients who were randomised to a follow-up schedule of clinical examination plus mammography or to a more intensive follow-up schedule of laboratory tests combined with radiological imaging. The protocols for these are shown in Tables 1 and 2[6-8].

**Table 1:** Randomised trial of intensive schedule versus standard schedule of follow-up of patients in the "GIVIO" trial

**Intensive follow-up (n = 655)**	**Standard follow-up (n = 665)**
• Physical examination every 3 months for 2 years; then 6monthly for 3 years	• Physical examination every 3 months for 2 years; then 6monthly for 3 years
• Serum biochemistry at each clinical examination	• Mammography annually
• Chest x-ray every 6 months	
• Isotope bone scan annually	
• Liver ultrasound annually	
• Mammography annually	

(study detailed in JAMA 1994; 271: 1587–1592)

**Table 2:** Schedule for follow-up in the roselli del turco trial of intensive follow up

**Intensive follow-up (n = 622)**	**Standard follow-up (n = 621)**
• Physical examination every 3 months for 2 years; then 6monthly for 3 years	• Physical examination every 3 months for 2 years; then 6monthly for 3 years
• Chest x-ray every 6 months	• Mammography annually
• Isotope bone scan every 6 months	
• Mammography annually	

(JAMA 1999; 281; 1586 and JAMA 1994; 271: 1593–1597)

When the results of these trials were pooled together and examined there was found to be no significant difference in the five-year disease-free survival or overall survival for either group of patients [9]. However, there was a difference in the detection of asymptomatic metastatic disease. In patients followed-up intensively 31% had asymptomatic metastases compared with only 21% in those being less intensively followed-up. Therefore, although an intensive follow-up schedule will detect metastatic disease earlier it does not impact on the patients' outcome with respect to disease-free and overall survival. In terms of quality of life, there were no differences between the patients in having intensive or less intensive follow-up schedules.

Furthermore, a recent systematic review and meta-analysis has focused on whether routine hospital visits were even effective in detecting loco-regional recurrences in patients who were asymptomatic following treatment for early breast cancer [10]. A total of 5,045 patients from 12 studies were analysed. It was found that in asymptomatic patients, only 40% of isolated loco-regional recurrences were diagnosed by routine visits and tests [10] but the difficulties with interpretation of the studies due to their poor quality was also clear from this analysis. However, the majority of recurrences were identified outside the patients planned routine follow-up schedule.

Another question that is now being asked, particularly in view of the lack of oncologists in many areas in the world, is what is the value of patients attending a "specialist" follow-up clinic? In a trial designed to address this question, after treatment for breast cancer 296 patients were randomised to follow-up, either by their general practitioner or by hospital specialist [11]. Although this was a small study, it was stated that there was no significant difference in the detection of metastases in the two groups of patients. However, there was a 60% increased detection in the group of patients being followed-up by the hospital specialists, although this was dismissed because it did not achieve statistical significance. Another important aspect of the findings from this study was that there was no difference in the patients' quality of life. Furthermore, patients in the general practitioner group were more satisfied with the continuity of care than patients' follow-up by hospital specialists. The limitations of this study, particularly in respect of the statistical power and short period of follow-up do limit the conclusions that can be drawn. Furthermore, one third of eligible patients declined to participate in this study. A further well designed and larger clinical study is necessary to address this issue.

An alternative approach to the follow-up was evaluated in a Swedish multicentre study where the role of nurse-led follow-up was examined [12]. A small group of 264 patients with early breast cancer were randomised to routine physician follow-up or follow-up by a nurse specialist. There was no difference in terms of patient satisfaction, anxiety, or depression and there were no differences between time to recurrence or death between the two groups of patients. The authors did note that the study was small and not powered to detect differences in recurrence and survival. On the basis of these encouraging results further studies would be required to confirm that this is an alternative way of follow-up for certain selected patients which may offer advantages in terms of continuity of care, patient education and allow a more appropriate utilisation of physician-time [12].

A key consideration is what do the patients themselves want? One small, randomised trial of 211 patients has addressed this issue [13]. Patients were randomised to have either a conventional follow-up clinic visit schedule (every 3 months for the first year, four months for the second year, six months up until 5 years after initial diagnosis and annually thereafter) or just to have mammography at routine intervals (initially every year for 5 years, then two yearly). Those patients who had undergone a mastectomy had a slightly different mammographic schedule with a mammogram one year after diagnosis and then every two years subsequently [13].

The results of this study revealed that approximately twice as many patients felt they would rather have a reduced schedule of follow-up rather than a more intensive one. Also the patients in the groups randomised to just mammographic follow-up were satisfied with this. However, a very important point to emerge was that this was not a universal finding amongst all the patients in the study. Importantly, those patients who were less than 50 years of age, who were at a stage between two and five years after diagnosis and those who had aggressive disease, were less likely to participate in this study [13].

### Just how do we follow-up patients and what are the guidelines?

Given the data from this small number of randomised trials is it possible to decide upon, in an evidence-based fashion, the most appropriate follow-up for patients with breast cancer? The key facts to consider in attempting to do this and which have emerged from these randomised controlled trials are:

• 25% of patients develop recurrence and in 60% to 80% of them this will be in the three years following initial diagnosis,

• Hospital outpatient visits may detect 40% of locoregional recurrences in asymptomatic patients,

• A very intense schedule of hospital visits, laboratory and imaging tests does not affect disease-free or overall survival compared with a less intense schedule of clinical examination and mammography,

• Follow-up in hospital clinics compared with general practice follow-up does show a trend towards an increased detection of metastatic disease but quality of life was no different although the longer term effects on survival are unclear

• Patients' preference is for less intense follow-up with the exception of younger patients, those with more aggressive disease and those who are at a stage of between two to five years from their initial diagnosis.

With these considerations in mind, clinical practice guidelines which have been developed and defined as "systematically developed statements to assist practitioner and patient decisions about appropriate health care for specific clinical circumstances" [14], have been developed for the follow-up of patients with breast cancer. These guidelines have been developed on the basis of systematic reviews of the literature and in each guideline there is usually an explanation of the classification of levels of evidence (e.g. from meta-analyses, systematic reviews of RCTs, case-control studies, cohort studies, non-analytic studies and expert opinion), classification of grades of recommendation (e.g. based on the results from meta-analyses of RCT, or from a high quality RCTs, or from case control or cohort studies, or based on non-analytical studies or expert opinion) and also the guideline will state the time when it is due to be updated.

In recent years in the United Kingdom, a number of different guidelines have been produced in an attempt to ensure that patients with breast cancer have the most appropriate follow up. For example, the Association of Breast Surgery at the British Association of Surgical Oncology (BASO), the Scottish Intercollegiate Guidelines Network (SIGN), and the National Institute of Clinical Excellence, the Royal College of Radiologists and the Clinical Outcomes Group of the Department of Health have all tried to provide guidance as to the most appropriate way of follow-up [15-19]. The key points to emerge from these guidelines are shown in Table 3, and have their basis in the data outlined above. In addition to these guidelines from the UK, a variety of other organisations throughout the world have also produced their own guidelines, which consider follow-up of patients with breast cancer. It is beyond the scope of this article to consider these in detail but can be accessed elsewhere [20]. However, most recommend clinical examination every 3 to 6 monthly for 3 to 5 years, and then followed by annual clinical examinations. As regards mammography, the trials addressing this seems to recommend mammography six months after completion of radiotherapy and then at 1 or 2 yearly intervals thereafter.

**Table 3:** Guidelines issued in the united kingdom for the follow-up of patients with breast cancer

**Organisation**	**Recommendation**
The Association of Breast Surgery at the British Association of Surgical Oncology	• Patients on active treatment may be followed up until such treatment has been completed • High risk patients may be followed up more closely with joint care by surgeons and oncologists according to local protocols • Data about long term follow-up is essential in monitoring clinical outcomes • Patients to be followed up for 5 years • Routine mammography every 1 to 2 years for 10 years after diagnosis
Scottish Intercollegiate Guidelines Network (SIGN)	• These guidelines state that there is insufficient clinical evidence to determine the optimal interval of clinical examination. They suggest that a "pragmatic schedule" should be adopted, for example, every 6 months for 2 years and then annually thereafter. • For mammographic follow-up, in a breast which has been conserved, then this should be performed at least every 2 years and at intervals of not less than 1 year. For the contralateral breast mammography should be carried out every 1 – 2 years.
National Institute for Clinical Excellence (NICE)	• Guidelines state that there should be a "limited" follow-up for 2 – 3 years and should be agreed by "local networks". This would not normally exceed 3 years unless patients were in clinical trials. • The guidelines state that local networks should agree evidence-based policy for the frequency of mammographic follow up
The Royal College of Radiologists	• Guidelines recommend that mammography is carried out at least every 2 years and not more than annually
The Clinical Outcomes Group, Department of Health	• Recommends that mammography is carried out annually for 5 years and then every two years after that

It is always difficult for clinicians, particularly when several different guidelines exist in one county. However, it is easier to consider the areas common to these guidelines initially. All the guidelines agree that follow-up should be limited to clinical examination and mammographic surveillance with their being no recommendations for other laboratory or radiological imaging tests. But what is the interval as which these should be carried out and for how long? The difficulty is, of course, that there is no evidence on which to make these recommendations.

However, perhaps a clinical examination every six months for five years would be a reasonable recommendation but with the caveat of the lack of information available. Even more difficult to base on scientific evidence is the optimal interval for mammographic surveillance. Again we do not have the evidence but we must consider the randomised trials, what we understand about the biology of breast cancer and recurrence, and what we know from the various breast screening programmes that have been implemented internationally. Given this data an annual mammogram for five years and then every two years after breast conserving surgery seems a reasonable compromise. In patients who have had a mastectomy, then a surveillance mammogram every two years also seems to be a reasonable compromise for follow-up in these patients. However, we emphasise the lack of evidence for recommendations regarding mammographic surveillance.

This still leaves two important questions as to how long the patients should be followed-up for and by whom? Firstly, in terms of duration of follow-up, although we do not have the evidence, the National Institute of Clinical Excellence (NICE) recommends a limited period of follow-up of 2 to 3 years unless patients are entered into clinical trials. NICE also documents the financial savings that would accrue if such a policy were adopted [17]. However, there are many unanswered questions as to the appropriateness of this that needs to be answered. As regards who should follow-up the patient, one trial indicated that in selected patients this could be the general practitioner, but it is worth noting important exceptions as discussed above. Furthermore, the longer-term consequences of this approach remain unclear.

Another complicating factor with regard to follow-up is that several recently published trials have had a major impact on the usage of adjuvant hormone therapy, eg the indication that the armomatase inhibitors may be superior to tamoxifen, the value of further treatment with letrozole after 5 years of tamoxifen and the impact of changing patients after two or three years tamoxifen treatment to an aromatase inhibitor. Therefore, at the present time we are still not sure what the optimum for adjuvant hormonal therapy is and we also need to consider what should happen to the patients who are currently taking tamoxifen.

We also need to consider what will be the impact on bone mass with the increasing use of aromatase inhibitors and whether or not 'prophylactic' bisphosphonates may be required. Furthermore, intensive folow up does increase the rate of detection of asymptomatic metastases and whilst this did not impact on patients survival in the studies from the 20 years ago, with the current advances in systemic treatment (eg aromatase inhibitors, taxanes, trastuzumad etc) is it not possible that early treatment may now have survival advantages for these patients? One must not under estimate the need for continuing audit of results following the treatment for breast cancer and of the morbidity in these patients undergoing 'multi-modality therapy' which can have significant short and long term consequences. These are just some of the issues to be considered when following-up patients following treatment for breast cancer and therefore with increasing complexity of management it is likely that specialist input will be necessary still.

## Conclusion

Despite the advances that have been made in the treatment of patients with breast cancer we are still unclear as to the optimal way in which patients should be follow-up once treatment has been completed. Despite the publication of many different guidelines with recommendations for follow-up it is clear that the evidence base on which these are founded is lacking at the present time. Furthermore, the evidence is from trials that were commenced twenty years ago and their relevance to the modern management of patients with breast cancer is now questionable.

It is now important that we consider the research priorities in follow-up of breast cancer patients, in particular with respect to stratifying patients according to their risk of disease recurrence, and the impacts of treatments on physical and psychological morbidity and quality of life. It is essential that well designed randomised controlled trials are undertaken if we are to understand the most appropriate and effective ways of following up patients with breast cancer.

## Competing interests

**SDH **is a member of the national committee of the Association of Breast Surgery at the British Association of Surgical Oncology and in this regard was involved in the process of development of the Association of Surgery guidelines which are referred to in this paper

## Authors' contributions

**SDH **initiated the manuscript and the literature review.

**SDH, SC, AWH **and **TKS **drafted the manuscript and all authors approved the final manuscript
